# Associations between Attention-Deficit Hyperactivity Disorder (ADHD) Treatment and Patient Nutritional Status and Height

**DOI:** 10.1155/2018/7341529

**Published:** 2018-10-02

**Authors:** Mariana F. Granato, Alexandre A. Ferraro, Denise M. Lellis, Erasmo B. Casella

**Affiliations:** Pediatrics Department, School of Medicine, University of Sao Paulo, Av. Dr. Enéas Carvalho de Aguiar, 647, 05403.000, São Paulo, SP, Brazil

## Abstract

Attention-deficit hyperactivity disorder (ADHD) has been found to co-occur frequently with obesity, although the reasons for this association are unknown. The aim of this study was to compare the nutritional profile of a Brazilian cohort of ADHD patients with that of the general population and to analyze the association between ADHD drug treatment (with methylphenidate), nutritional status, and height of these individuals. In the first phase of the study, we designed the nutritional and height profile of 93 ADHD patients (5.1 to 13.8 years old) and compared it to a control group. In the second phase, we analyzed the association of the use of methylphenidate with nutritional status and height. The results showed that the prevalence of overweight/obesity was statistically higher in the cohort of ADHD patients compared to controls (40.9% vs. 34.7%; *P* < 0.05). After treating ADHD patients with methylphenidate, a statistically significant decrease in the BMI *z*-score was observed (0.695 vs. 0.305; *P* < 0.01). On the other hand, no significant impact on height was detected after treatment (0.189 vs. 0.248; *P* = 0.298). In conclusion, the results suggest that the use of methylphenidate in patients who have ADHD and obesity is relevant not only for controlling ADHD symptoms but also for improving the nutritional status of these individuals. Moreover, the treatment did not affect the patients' height.

## 1. Introduction

Attention-deficit hyperactivity disorder (ADHD) is the most common childhood behavioral disorder. Its estimated prevalence is 5.29% [1], though it varies with age. The mechanisms underlying ADHD are not fully understood; however, there is believed to be an interaction between genetic and environmental factors. Brain dopaminergic and noradrenergic dysfunction may also exist, especially in such regions like the prefrontal cortex, striatum, and cerebellum [2].

Comorbidities are frequent among ADHD patients. Approximately 67% may also present with oppositional defiant disorder, conduct disorder, anxiety, and mood disorders [3].

Additionally, several recent studies showed that ADHD is highly prevalent in obese individuals and vice versa [4–9]. In 2004, Holtkamp et al. [7] found that the average body mass index (BMI) of 97 boys with ADHD was significantly higher than that of a control group of children. Similarly, data from the “National Survey of Children's Health 2003” showed an increased prevalence of obesity among individuals with ADHD [5, 8]. Some studies have shown the inverse association. Agranat-Meged et al. [4] identified comorbid ADHD among 57.7% of 26 patients (8–17 years old) hospitalized for morbid obesity treatment. A systematic review and meta-analysis published in 2015 included a total of 728,136 individuals and found that the pooled prevalence of obesity was increased by about 70% in adults and about 40% in children with ADHD compared with those without ADHD [10].

Researchers thus began seeking to establish possible mechanisms to explain this correlation. One hypothesis is that obesity or associated factors, such as sleep disorders, could lead to ADHD development [11–13]. Another hypothesis is that ADHD promotes obesity, primarily through a lack of inhibitory control and planning [14–21]. Finally, some authors argue that the diseases' interaction is mediated by common pathophysiological mechanisms, such as *reward deficiency syndrome* [13, 20, 22, 23].

The association between ADHD and obesity is also important when considering ADHD treatment. Stimulant medications (e.g., methylphenidate and amphetamine compounds) are indicated for most cases. The most frequent side effects include decreased appetite, abdominal pain, headaches, irritability, and sleep disturbances. Rare side effects include weight loss, tics, social withdrawal, and emotional changes.

Recently, researchers have shown concern about the possible effects of long-term treatment on patient growth. Several studies reported a decline in growth rates after treatment onset in patients using stimulant medications for 2 or 3 years [24, 25]. In a 2008 review, Faraone et al. [26] reported that stimulant medications modestly but significantly delayed height growth and weight gain. They suggested that the growth deficit was dose-dependent and that medication suspension could promote normal growth recovery. In contrast, several studies showed no changes in the final height of patients after long-term medication use, as in the longitudinal case-control study conducted by Biederman et al. [27]. Their 10-year follow-up showed that stimulant medications did not influence patient height or weight gain.

Considering the impact of stimulant medication on nutritional status and height, it is meaningful to determine the effect of these medications on patients with ADHD and obesity. A longitudinal clinical trial published in 2009 followed 78 obese adults diagnosed with ADHD (65 received drug treatment for ADHD and 13 served as controls, since they did not accept or tolerate the ADHD treatment). After an average of 466 days of intervention, treated individuals weighed 12.36% less than their initial weight, while controls weighed 2.78% more than their initial weight [28]. Likewise, the meta-analysis conducted by Cortese et al. showed that, differently from the observed with unmedicated individuals, patients diagnosed and receiving pharmacological treatment for ADHD were not at a higher risk for obesity [10]. Such studies suggest that stimulant medications may be important for controlling ADHD symptoms and for improving nutritional status in patients with comorbid ADHD and obesity.

Thus, the objective of this study was to compare the nutritional profile of a Brazilian cohort of ADHD patients with that of the general population. Moreover, we also evaluated the influence of ADHD drug treatment on patient nutritional status and height.

## 2. Materials and Methods

This retrospective cohort study was divided into two stages. First, we compared the prevalence of overweight and obesity in ADHD patients with that in the general population. Then, we evaluated the association of methylphenidate use with the nutritional status and height of ADHD patients.

We identified all patients diagnosed with ADHD, according to the DSM criteria and classified in ICD-10 as F90.0 (*n* = 252; 206 boys [81.7%], 46 girls [18.3%]) registered in the Learning Disorders Clinic of the Children's Institute, Medical School of the University of Sao Paulo, since 2002. We surveyed their medical records for weights and heights before and after methylphenidate treatment.

We excluded patients (1) with comorbidities (e.g., genetic syndromes, hypothyroidism, diabetes mellitus, renal failure, and heart diseases) and those using other medications (e.g., neuroleptics, antipsychotics, antidepressants, and antiepileptics) that could interfere with nutritional status; (2) who did not tolerate methylphenidate; (3) who was already using methylphenidate or imipramine when follow-up was begun; and (4) who were dieting to gain or lose weight or who performed high-level physical activity during the treatment period. Additionally, individuals presenting incomplete medical records with respect to information related to weight, height, and use of medications were also excluded. Overall, 159 patients were excluded. The 93 patients remaining were included in the analysis. Twenty were girls (21.5%) and 73 were boys (78.5%), aged between 5 years and 1 month to 13 years and 9 months old (average, 9 years and 5 months; median, 9 years and 2 months).

For the first study phase, we recorded patients' weights and heights before they started drug treatment to avoid any interference with nutritional status.

The average time between the diagnosis of ADHD and the beginning of drug treatment was 3 months, and patients did not receive other types of treatment (such as behavioral therapy) during this period. The weight and height were measured by the Clinic of Learning Disorders physicians while patients were barefoot and wearing minimal clothing. We used digital anthropometric scales with 0.1 kg accuracy and wall stadiometers with 0.1 cm graduations. We calculated the BMI and BMI *z*-score for each patient using AnthroPlus (http://www.who.int/growthref/tools/en), which calculates the exact *z*-score (the “absolute” *z*-score), not only its range.

Using BMI *z*-score curves and tables developed by the World Health Organization, we classified the children's BMIs according to

- Thinness: *z*-score < −2 standard deviations
- Normal weight (eutrophy): *z*-score ≥ −2 and <1 standard deviation
- Overweight: *z*-score ≥ 1 and <2 standard deviations
- Obesity: *z*-score ≥ 2 standard deviations

Using these results, we developed a profile of ADHD children and adolescents before drug treatment. Moreover, we compared the patients' nutritional profiles with those of control group patients to evaluate whether the prevalence of overweight/obesity was higher than expected.

The control group consisted of children from another study of 1001 children, aged 2–14 years, who received pediatric emergency care between March 2009 and April 2010. To avoid confounding variables, we excluded individuals presenting with diarrhea and/or vomiting and chronic diseases, in poor condition, and/or individuals whose symptoms significantly worsened during treatment. The two populations were quite similar, since both comprised children and adolescents living in Sao Paulo who regularly used the public health system. Further, we included only patients in the same age group (5–14 years). The individuals were randomly selected to achieve an equivalent sex distribution between the study and the control groups.

In the second study phase, BMI and height changes during methylphenidate treatment were determined to evaluate the association between drug treatment, nutritional status, and height. We compared pre- and posttreatment absolute *z*-scores for both BMI and height.

### 2.1. Statistical Analysis

We analyzed nutritional profile differences between patients and controls with the chi-squared test. To evaluate the associations between methylphenidate use and patient height and nutritional status, we analyzed pre- and posttreatment *z*-scores for BMI and height with the paired Student *t*-test.

### 2.2. Ethical Aspects

This was a retrospective analysis that did not expose included individuals to experimental intervention. We received institutional review board approval, and project expenses were financed by the authors, who had only scientific interest in the study.

## 3. Results

The analysis of the data of the first phase of the study indicated a significant difference in the prevalence of overweight/obesity between the ADHD and the control groups (40.86% vs. 34.73%; *P* = 0.0147), as shown in Table 1.

The subsequent analysis of the association between methylphenidate treatment and nutritional status and height in ADHD patients included the same 93 patients used in the first study phase.

All patients were treated only with methylphenidate. The end of treatment was defined as the time at which medication was suspended or at which another drug was prescribed that could have interfered with the nutritional status (e.g., risperidone, valproic acid, clonidine, fluoxetine, and sertraline), generally due to comorbidities. The average treatment duration was 2.6 years (median, 2.1). Thirty-two patients (34.4%) were treated for >3 years, to a maximum of 8.6 years.

The average pretreatment BMI absolute *z*-score was 0.695. The average posttreatment BMI absolute *z*-score was 0.305. The paired Student *t*-test revealed that methylphenidate significantly decreased the BMI *z*-score (*P* < 0.01). We obtained similar results for patients treated longer (>3 years): the average pretreatment *z*-score was 0.405, and the average posttreatment *z*-score was −0.210 (*P* < 0.01) (Table 2).

There were no significant differences between pre- and posttreatment absolute height *z*-scores (*P* = 0.30). The average pretreatment height *z*-score was 0.189, whereas the posttreatment height *z*-score was 0.248.

Because several studies have suggested that medication use may more significantly affect height during the first years of treatment with a tendency for effects to decrease over time, we separately analyzed patients that used the medication < 1 year and those who used it < 3 years and >3 years, with no significant differences observed (Table 3).

We also analyzed the evolution of BMI classification posttreatment. Of 10 obese patients (pretreatment), 4 (40%) became overweight and 6 (60%) remained obese posttreatment. Of 28 overweight patients, 1 (3.6%) became obese, 14 (50%) remained overweight, 12 (42.9%) became normal weight, and 1 (3.6%) became thin. Of the 55 normal-weight patients, 6 (10.9%) became overweight, 2 (3.6%) became thin, and 47 (85.5%) remained normal weight.

**Figure 1:**
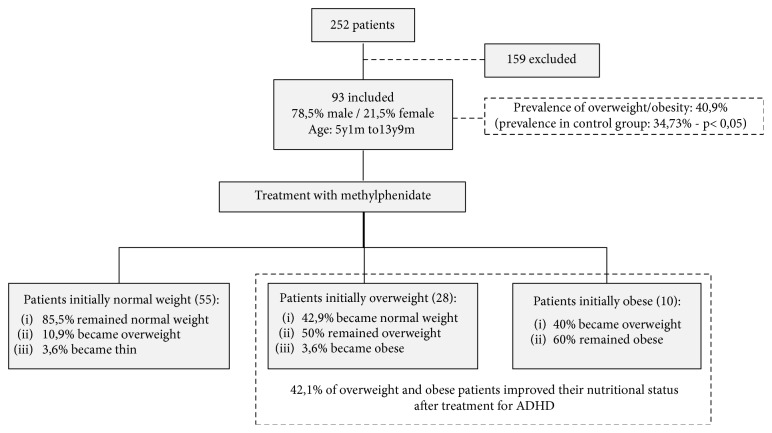
Study design and results.

The study design and the synthesis of the main results are shown in Figure 1.

## 4. Discussion

Many recent studies have shown an increased prevalence of ADHD in obese individuals and vice versa, and researchers have sought to elucidate the mechanisms underlying this association.

A possible explanation is that obesity or associated factors, such as sleep fragmentation and hypoxemia caused by apneas and hypopneas, might contribute to daytime sleepiness, explaining inattention symptoms compatible with ADHD [11, 12]. Similarly, Weinberg and Brumback proposed the “*hypoarousal theory*,” which suggests that ADHD individuals feel sleepier than controls do and may manifest hyperactivity and impulsivity to remain awake and alert [13]. A study using the multiple sleep latency test (MSLT) confirmed that children with ADHD are sleepier than controls [29].

In contrast, some researchers hypothesize that ADHD contributes to obesity because the lack of inhibitory control, typical of ADHD patients, could result in a lack of planning and difficulty in self-monitoring behavior, as it occurs in the loss of control eating episodes [30]. Similarly, delay aversion, common among ADHD patients, might encourage the consumption of high-calorie foods, such as fast food, instead of healthier, home-cooked foods [16, 21]. Inattention and executive dysfunction may also hinder regular diet patterns, since patients may have a low perception of satiety signals [14, 15, 17, 18]. Another potential pathophysiological mechanism recently described is the hypocretin/orexin system, composed by hypothalamic peptides involved in the control of a variety of physiologic mechanisms, including sleep/alertness, feeding behavior, and endocrine and autonomic functions. Some authors speculate that patients with ADHD have an alteration in this hypocretin/orexin system, which could lead to deficits of alertness and abnormal eating behaviors [31]. Moreover, ADHD patients have difficulties in dealing with certain emotions and are often exposed to frustrating situations, which may lead them to overeat as an escape mechanism [16, 19–21].

Finally, some authors argue that the interaction between obesity and ADHD is mediated by common pathophysiological mechanisms. One mechanism with great explanatory potential is the “reward circuit.” “Reward pathways” are essential for survival, since they allow feelings of pleasure during feeding and reproducing, the so-called “natural rewards,” through the release of neurotransmitters, especially dopamine, in the nucleus accumbens and frontal lobes. However, dopamine can also be released by “nonnatural rewards,” such as alcohol, drugs, compulsive activities (like gambling), and other risky behaviors. Some authors suggest that individuals with disabilities in the “natural action” of neurotransmitters may develop “reward deficiency syndrome” and thus become more susceptible to abusing “nonnatural rewards” [2]. As previously noted, reduced brain dopaminergic activity plays a central role in ADHD pathophysiology, predisposing these individuals to reward-deficiency syndrome. As it occurs in alcohol and drug abuse, high-calorie food consumption can activate dopaminergic pathways. Therefore, overeating might be a form of self-medication for ADHD patients to compensate, at least temporarily, for dopamine deficits [16, 22, 23, 32].

In this study, we observed a higher prevalence of overweight/obesity in ADHD patients compared to that in controls. The proportion of obese individuals was lower in the study group (10.75% vs. 17.66%), but the prevalence of overweight individuals was much higher (30.11% vs. 17.07%). Additionally, no ADHD patients were thin pretreatment, while 2.10% of controls were (*P* < 0.05). This finding corroborates the results of previous studies showing an increased prevalence of overweight/obesity in ADHD individuals [5, 7, 8, 10].

We obtained very interesting results regarding the associations between methylphenidate, nutritional status, and height. First, absolute BMI *z*-scores were significantly reduced during treatment. The average pretreatment BMI *z*-score was 0.695, which dropped to 0.305 (*P* < 0.01). It is widely known that methylphenidate can reduce appetite, leading to weight loss [33]. However, this effect is generally temporary and becomes less evident as treatment progresses. To assess whether treatment duration influences weight loss, we compared patients treated <1 year (26 patients) with those treated >3 years (32 patients). BMI in both groups tended to decrease, with statistically significant variation, indicating that BMI tended to decrease regardless of the treatment period. However, the group of patients treated for >3 years had a negative average BMI *z*-score posttreatment (−0.210). Since the anorectic effects of methylphenidate are usually transient and become less evident during treatment, this mechanism may not totally explain weight loss in all situations, especially in individuals treated >3 years. Accounting for the mechanisms suggested by Davis et al. [16] and Strimas et al. [21], medication also likely reduced impulsiveness and inattention and improved inhibitory control, leading to lower caloric intake and a more organized dietary pattern, which may have helped overweight/obese individuals lose weight.

A separate analysis showed that 42.1% of overweight and obese patients improved their nutritional status after treatment for ADHD: 42.9% of overweight patients became normal weight, and 40% of obese patients became overweight. These results suggest that methylphenidate helps control inattention and hyperactivity/impulsivity as well as weight in patients with comorbid ADHD and obesity, similar to results from Levy et al. [28] and from the meta-analysis from Cortese et al. [10].

This weight loss could be worrisome for normal-weight patients starting treatment, as the medication could lead to a weight deficit and malnutrition. However, the vast majority (85.5%) of normal-weight individuals in this study remained eutrophic. Only three individuals overall (3.2%) became thin, with a minimum *z*-score of −2.81.

We observed no statistically significant difference in height *z*-scores of patients receiving drug treatment. The pre- and posttreatment average height *z*-scores were 0.189 and 0.248, respectively. Since some authors hypothesize that the growth-reducing effects of stimulants would become less evident over time and thus no significant impact would be observed in the final height of the individual, we separately evaluated patients treated <1 year (26 patients), <3 years (61 patients), and >3 years (32 patients). In the subgroups, no statistically significant difference was observed in the final height *z*-score when compared to the initial value. Unlike observations by Faraone et al. [26], individuals treated for shorter periods (<1 year or <3 years) showed no growth rate reductions.

These data are consistent with the findings of Biederman et al. [27] and Peyre et al. [34], in which no impact of drug treatment on patients' height was observed. As in studies from other countries, we observed a higher prevalence of overweight/obesity in ADHD patients from among a Brazilian cohort of children and adolescents. Moreover, the results suggest that for ADHD patients with comorbid obesity, methylphenidate treatment is important not only for controlling ADHD symptoms but also for reducing BMI and, subsequently, for improving their nutritional status. The drug also does not seem to impair growth and does not significantly change the nutritional status of normal-weight individuals.

### 4.1. Strengths and Limitations of the Study

Regarding study strengths, we aimed to eliminate possible confounding variables by excluding patients who used other medications that could affect the nutritional status as well as patients with comorbidities that could have influenced the results. Using a control group comprising individuals matched for sex and age group and presenting similar socioeconomic characteristics as the patient group is another strength. In addition, we only studied methylphenidate, which renders our results quite consistent.

Regarding limitations, the study was retrospective, which reduces its impact. Additionally, weight and height were not measured by the same professional, and different equipment was used. This could have led to slight variations in anthropometric measurements. However, we believe that any variations did not significantly affect the results, since weight and height measurements are objective and routinely performed by physicians.

Another limitation of the study is that the proportion of boys and girls found in the sample of ADHD patients differs significantly from that observed in the population. In our sample, we had 3.6 boys : 1 girl, while in the population this proportion is around 2.5 boys : 1 girl [3].

## 5. Conclusions

These results are promising and consistent with those from previous studies, suggesting a possible association between ADHD and obesity. Because of the study design, it is not possible to establish a causal relationship between different disorders, only an associative one.

We conclude the following:

- The prevalence of overweight/obesity observed in ADHD patients was significantly higher than that in the general population
- No association between methylphenidate and height was observed, regardless of treatment duration
- In the study sample, the use of methylphenidate seems to be related to a reduction in the BMI *z*-score. This reduction was more significant in overweight/obese individuals, suggesting that in patients with comorbid ADHD and obesity, drug treatment is important not only for controlling ADHD symptoms but also for reducing BMI and subsequently improving the nutritional status of these individuals
- Most normal-weight individuals treated with methylphenidate remained eutrophic, suggesting that this drug does not significantly reduce the weight of these individuals

## Figures and Tables

**Table 1:** Nutritional profiles of the study sample and the control group.

Nutritional status	Study sample			Control group		
	Male	Female	Total	Male	Female	Total
Thin and normal weight	42 (57.53%)	13 (65.00%)	55 (59.14%)	172 (65.65%)	46 (63.89%)	211 (65.27%)
Overweight and obese	31 (42.47%)	7 (35%)	38 (40.86%)	48 (34.35%)	26 (36.11%)	116 (34.73%)
Total	73	20	93	262	72	334

Comparisons between groups were made by the chi-squared test. The significance was set at *P* < 0.05. Data are presented as *n* (%).

**Table 2:** Comparison between pre- and posttreatment BMI *z*-scores.

	Average pretreatment *z*-score	Average posttreatment *z*-score	Statistical analysis (Student *t*-test)
Total sample (*n* = 93)	0.695	0.305	*P* < 0.01
Treatment < 1 year (*n* = 26)	0.985	0.747	*P* < 0.01
Treatment < 3 years (*n* = 61)	0.847	0.575	*P* < 0.01
Treatment > 3 years (*n* = 32)	0.405	−0.210	*P* < 0.01

BMI: body mass index.

**Table 3:** Comparison between pre- and posttreatment height *z*-scores.

	Average pretreatment *z*-score	Average posttreatment *z*-score	Statistical analysis (Student *t*-test)
Total sample (*n* = 93)	0.189	0.248	*P* = 0.298
Treatment < 1 year (*n* = 26)	0.276	0.360	*P* = 0.068
Treatment < 3 years (*n* = 61)	0.341	0.429	*P* = 0.107
Treatment > 3 years (*n* = 32)	−0.10	−0.10	*P* = 0.97

## Data Availability

The data and statistical analysis used to support the findings of this study are available from the corresponding author upon request.

## Supplementary Materials

Supplementary Materials The supplementary material submitted consists of the database and statistical analyses used to support the findings of the study, as oriented on the data sharing policy of the journal.
